# Supplementary material for: Architecture for sub-100 ms liquid crystal reconfigurable intelligent surface based on defected delay lines
Source: Commun Eng. 2024 May 18;3:70. doi: 10.1038/s44172-024-00214-3 (PMC11102442; doi:10.1038/s44172-024-00214-3)
Supplement: Supplementary file 2 — Supplementary Information [file 44172_2024_214_MOESM2_ESM.pdf]

Supplementary information: Architecture for sub-100 ms Liquid Crystal Reconfigurable Intelligent Surface Based on Defected Delay Lines

Robin Neuder[1], Marc Späth[1], Martin Schüßler[1], Alejandro Jiménez-Sáez[1]

[1]Institute of Microwave Engineering and Photonics, Technical University of Darmstadt, Merckstraße 25, Darmstadt, 64283, Hessen, Germany

Characterization of delay line featured in RIS_5

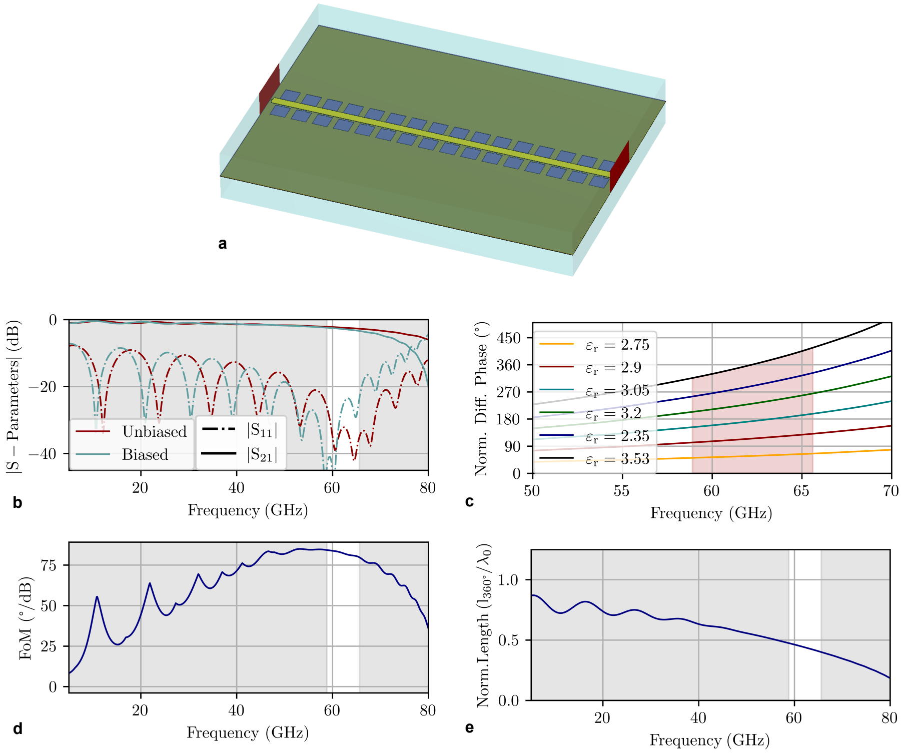

Supplementary Figure 1: **Simulated tunable delay line characterization for RIS_5**. (a) Simulation model of the delay line with a Liquid Crystal layer thickness of 4.6 μm. (b) Simulated absolute transmission and reflection coefficients of the delay line. (c) Simulated differential phase shift in the tunable delay line for increasing relative permittivity of the Liquid Crystal. The red area marks all achievable differential phases. (d) Simulated Figure of Merit of the delay line and (e) simulated Normalized Length of the delay line.

Measured bandwidth of RIS_5

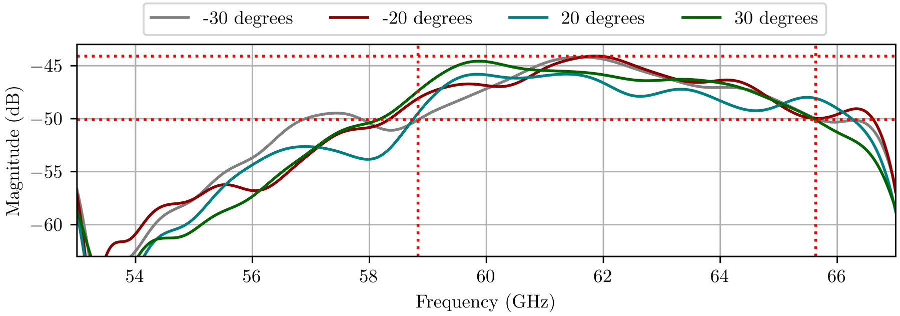

Supplementary Figure 2: **Measured bandwidth of RIS_5**. Measured $-6\,\mathrm{dB}$ bandwidth of RIS_5 for steering angles towards -30°, -20°, 20° and 30°. The $-6\,\mathrm{dB}$ bandwidth is measured from the point where the received power is the strongest across all measurements. Frequency limits are selected as the inner bounds of all four measurements.

Fabricated sample of RIS_{10}

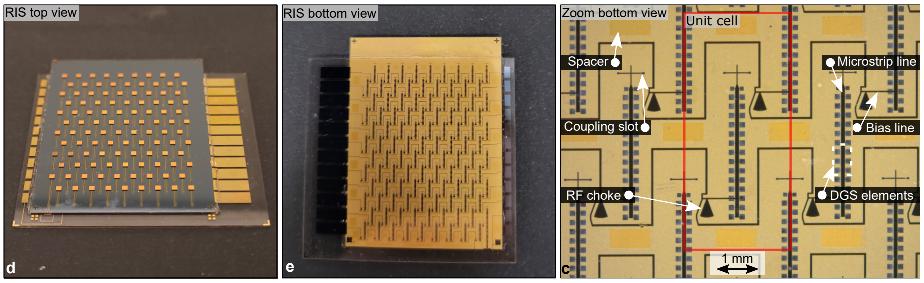

Supplementary Figure 3: **Fabricated sample of RIS_{10}**. (a) Top view and (b) bottom view of fabricated RIS_{10}. (c) Zoom in on bottom view of RIS_{10}. The red box indicates unit cell.

Dimensions of RIS_5 and RIS_{10}

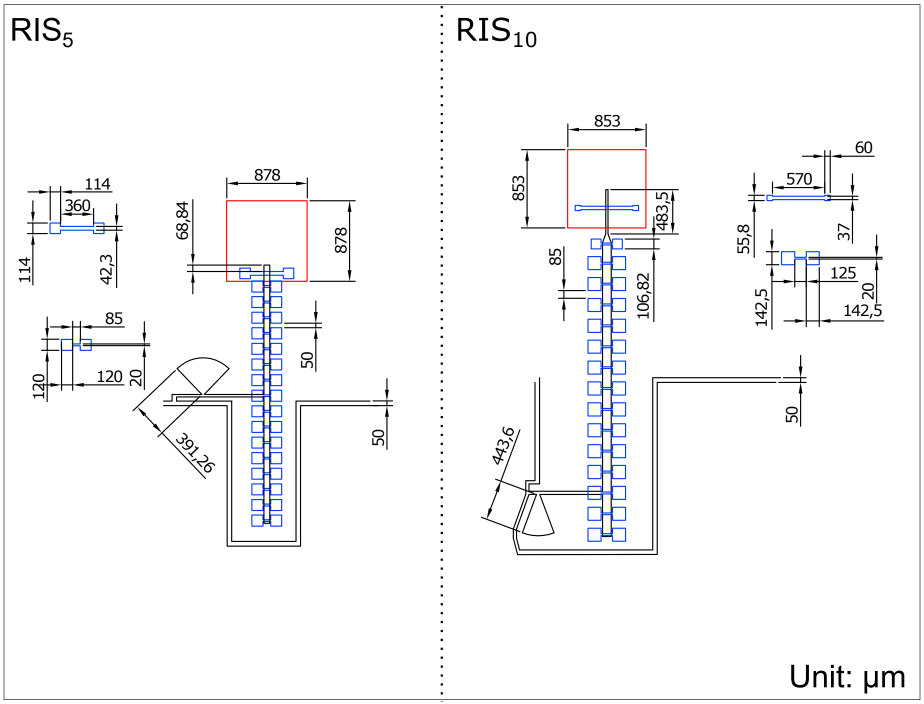

Supplementary Figure 4: **Dimensions of RIS_5 and RIS_{10} including tunable delay lines, RF-DC decouplers and radiating elements.**

Bistatic measurement results of RIS_{10}

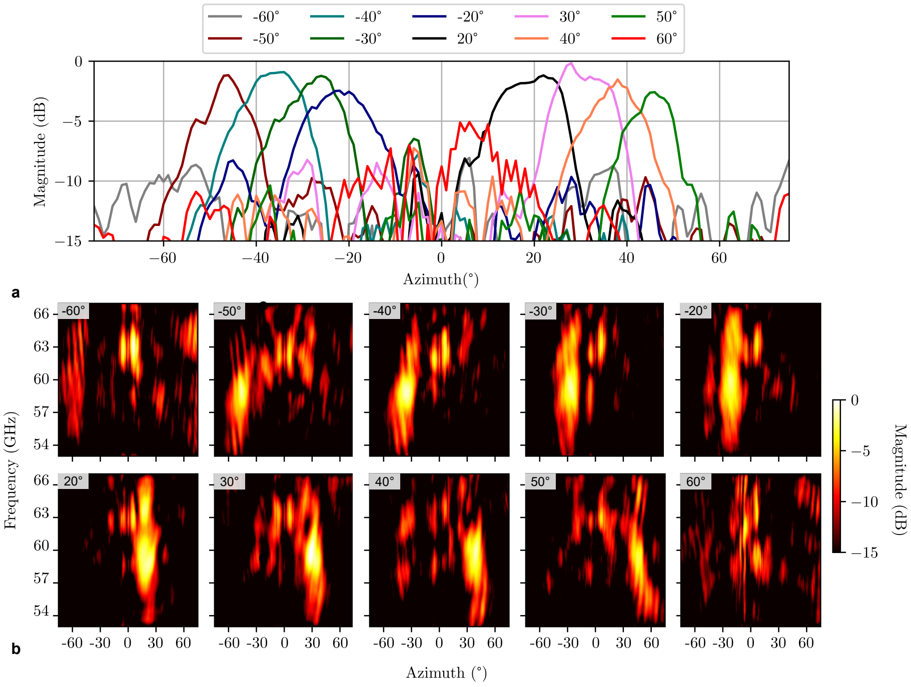

Supplementary Figure 5: **Bistatic measurements results of $\mathbf{RIS_{10}}$.** (a) Measured normalized response of RIS_{10} over azimuth at a frequency of 59 GHz for different voltage configurations. (b) Heat maps, illustrating the measured normalized broadband response of the RIS for desired steering angles between $-60°$ to $-20°$, and $20°$ to $60°$, respectively.

Supplementary Table 1: Voltages set for the bistatic measurements of RIS_5.

Angle(°)	Row 1	Row 2	Row 3	Row 4	Row 5	Row 6	Row 7	Row 8	Row 9	Row 10	Row 11	Row 12
-60	0	4.58	2.36	6.56	2.93	0.64	4.41	2.07	8.05	2.47	1.08	5.39
-50	0	5.32	2.98	17.66	3.65	2.21	10.64	3.66	1.95	8.23	2.14	2.24
-40	0	4.74	3.51	1.59	6.43	3.33	2.31	16.09	4.63	2.4	1.34	5.75
-30	0	5.26	4.16	2.75	1.11	7.79	4.83	3.09	2.21	16.74	3.19	3.06
-20	0	6.21	5.7	3.45	2.9	2.17	1.05	11.41	6.07	3.46	1.71	2.24
20	0	2.29	2.62	3.44	4.05	5.85	10.85	0.84	2.44	2.85	2.84	3.76
30	0	2.44	3.53	4.43	8.83	1.48	2.75	3.99	7.46	0.22	1.66	2.96
40	0	2.65	4.57	7.82	1.75	2.92	5.24	0.3	2.96	4.0	4.06	2.25
50	0	3.01	5.28	0.48	2.55	4.99	0.81	3.1	5.73	1.29	1.0	6.33
60	0	4.07	8.25	2.26	4.0	8.92	2.86	4.21	0.67	3.34	4.54	3.81

Note: All voltage values are provided in V_{pp}.

Supplementary Table 2: Voltages set for bistatic measurements of RIS_{10}.

Angle(°)	Row 1	Row 2	Row 3	Row 4	Row 5	Row 6	Row 7	Row 8	Row 9	Row 10	Row 11	Row 12
-60	0	5.03	1.15	4.4	2.71	4.06	2.57	5.64	2.7	4.51	2.86	17.79
-50	0	2.95	1.52	9.05	3.35	18.09	3.54	2.45	4.77	2.99	15.4	4.09
-40	0	3.45	2.6	20.48	5.12	2.83	17.1	4.36	2.92	15.84	4.32	3.28
-30	0	3.81	3.05	0.24	14.66	3.9	3.37	2.81	5.81	3.82	2.98	2.67
-20	0	4.27	3.26	8.26	3.09	2.2	5.73	4.41	3.52	2.92	2.49	0.66
20	0	1.78	2.47	1.8	5.75	5.77	2.34	2.96	3.38	3.65	4.74	6.9
30	0	2.29	3.36	5.9	1.21	2.81	3.91	5.94	2.18	2.87	3.75	4.65
40	0	3.06	4.14	0.31	3.81	4.63	0.57	3.23	4.58	0.93	2.88	4.13
50	0	3.77	6.59	1.67	6.18	0.26	3.21	6.68	2.68	3.54	0.51	3.12
60	0	3.17	9.38	2.44	4.94	1.96	3.76	2.54	4.42	0.97	4.52	0.01

Note: All voltage values are provided in V_{pp}.
